# Effects of Traditional Chinese Medicine Adjuvant Therapy on the Survival of Patients with Primary Liver Cancer

**DOI:** 10.1155/2022/9810036

**Published:** 2022-03-17

**Authors:** Jie Hou, Ke Shi, Yao Liu, Jialiang Chen, Chongping Ran, Xianbo Wang

**Affiliations:** ^1^Department of Gastroenterology, Dongzhimen Hospital, Beijing University of Chinese Medicine, Beijing, China; ^2^Center of Integrative Medicine, Beijing Ditan Hospital, Capital Medical University, Beijing, China

## Abstract

**Aim:**

This study aims to evaluate whether adjuvant traditional Chinese medicine (TCM) can improve the survival of patients with primary liver cancer (PLC).

**Methods:**

A total of 1,859 patients with PLC at Beijing Ditan Hospital between August 2008 and September 2017 were included. The patients were divided into TCM and control groups according to whether the patients took TCM for ≥3 months. There were 1,111 patients in the TCM group and 748 in the control group. Univariate and multivariate Cox regression analyses were used to analyze the factors affecting the 3-year survival of patients with PLC. To reduce selection bias, 1 : 1 propensity score matching (PSM) was performed between the two groups. The overall survival outcomes were evaluated using the Kaplan–Meier (K–M) survival curve, and the log-rank test was used to compare the differences in survival curves.

**Results:**

After multivariate Cox regression analysis, TCM was an independent favorable factor for the 3-year survival of patients with PLC (adjusted hazard ratio (aHR) 0.359, 95% confidence interval (CI) 0.292–0.441, *P* < 0.001). Before and after PSM, the 3-year overall survival rates were 33.3% and 54% in the control group and 79.7% and 69.7% in the TCM group, respectively. The 3-year mortality risk in the TCM group was lower than that in the control group for different PLC subgroups.

**Conclusions:**

TCM adjuvant therapy increased the 3-year overall survival rate of patients with PLC.

## 1. Introduction

Primary liver cancer (PLC) is a malignant tumor that originates from hepatocytes or intrahepatic bile duct epithelial cells [1]. It is currently the fourth most common cause of death from malignant tumors and the sixth most common malignant tumor in the world [2]. East Asia has the highest burden of hepatic carcinoma incidence and deaths [3]. In China, PLC is currently the fourth most common cancer and the second leading cause of death from tumors [4,5]. At present, the main treatment methods for PLC include surgical resection, radiofrequency ablation (RFA), transcatheter arterial chemoembolization (TACE), chemotherapy, and molecular-targeted drug therapy. With the development of modern medical technology, the prognosis of PLC has improved to a certain extent. However, the mortality rate remains high. A previous study showed that the 3-year overall survival rate of patients with liver cancer in Asian countries is only 19% [6]. Lin et al. showed that the 3-year overall survival rate of patients with BCLC stage B after TACE treatment was only 37% [7]. The high incidence and mortality rates of PLC require effective treatment to improve the survival rate.

As an important part of complementary and alternative medicine, traditional Chinese medicine (TCM) has been widely used for various cancers, including liver cancer [8]. Recently, a study showed that the combination of antiliver fibrosis TCM could significantly reduce the risk of liver cancer in patients with hepatitis B cirrhosis [9]. In recent years, as an adjuvant treatment method, TCM can reduce the side effects of radiotherapy and chemotherapy, improve the quality of life, and prolong survival time [10–12]. TCM has advantages in preventing and treating PLC. However, there are few large-scale studies on the clinical efficacy of Chinese patent medicines and Chinese medicine prescriptions for liver cancer, and their application value remains to be verified. Based on liver function and clinical liver cancer staging, we observed the effect of auxiliary TCM on the 3-year survival of patients with PLC.

Therefore, this study aims to explore whether adjuvant TCM therapy can improve the survival rate of patients with PLC. We sought to perform an in-depth analysis of the role of TCM in different populations of PLC to provide more clinical evidence for TCM treatment.

## 2. Materials and Methods

### 2.1. Research Subjects

We retrospectively extracted the electronic medical record data of 7,523 hospitalized patients with PLC between August 2008 and September 2017 at the Beijing Ditan Hospital of Capital Medical University (Beijing, China). Patients diagnosed with PLC and aged between 18 and 80 years were included in this study. Patients with other malignant tumors, metastatic hepatic carcinoma, pregnancy or breastfeeding, <3 years of follow-up, and incomplete relevant clinical data were excluded. The follow-up date for this study ended in September 2020. Finally, 1,859 patients with PLC were included in this study.

**Figure 1:**
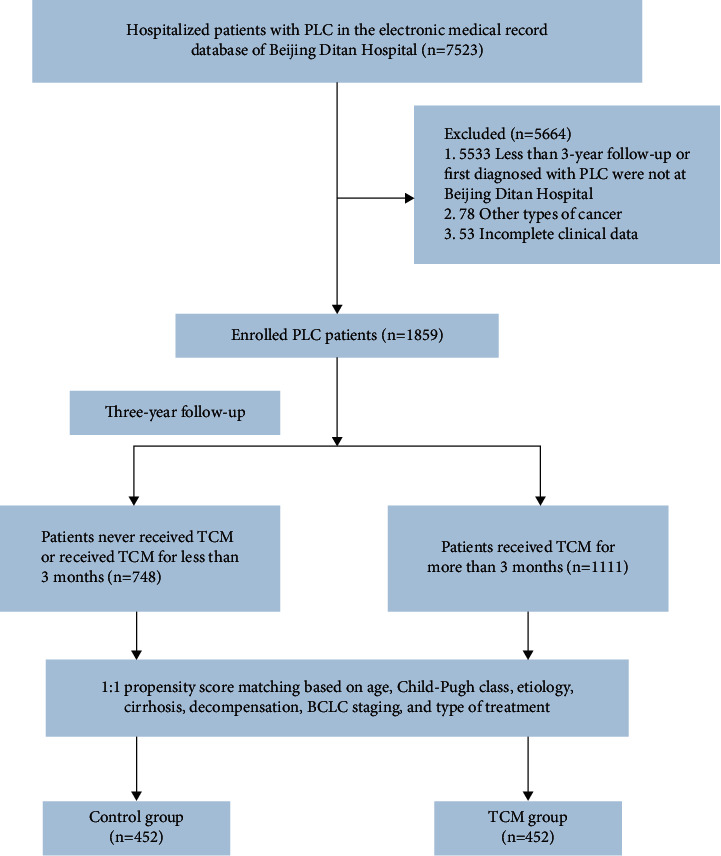
Flow chart for enrollment of patients with primary liver cancer.

To evaluate the impact of TCM treatment, all patients were divided into TCM and control groups according to whether patients took TCM cumulatively for ≥3 months during the follow-up period. Western medicine combined with TCM for ≥3 months (TCM group, *n* = 1111) or combined with TCM for <3 months (control group, *n* = 748). Western medicine treatment includes surgical resection; minimally invasive treatment, including TACE, RFA, microwave ablation, and percutaneous ethanol injection; and palliative treatment, including palliative symptomatic treatment, systemic chemotherapy, sorafenib, and lenvatinib. Based on the patient's age, Child–Pugh class, etiology, cirrhosis, decompensation, Barcelona liver cancer staging (BCLC), and type of treatment at the time of enrollment in this study, we performed 1 : 1 propensity score matching (PSM) between the two groups (Figure 1). The study was approved by the Ethics Review Committee of Beijing Ditan Hospital (Beijing, China) and complied with the ethical guidelines of the Declaration of Helsinki.

### 2.2. Clinical Definitions and Evaluation

The baseline date of this study was the date when the patient was diagnosed with PLC at the Beijing Ditan Hospital. Overall survival was defined as the time from the diagnosis of PLC to death or the 3-year follow-up time cutoff. Diagnosis of PLC through histopathological and/or imaging examinations was performed. Multiphase computed tomography or dynamic contrast-enhanced magnetic resonance imaging (MRI) showed typical lesions; namely, the arterial phase lesions were significantly enhanced, and the portal vein or delayed phase intrahepatic lesions had lower enhancement than the normal liver parenchyma. A diagnosis of liver cancer was established if one of the two methods demonstrated typical features in intrahepatic nodules ≥1 cm [13].

### 2.3. Study Medications

The nine compounds discussed in this study were approved by the State Food and Drug Administration (SFDA) of China. The national medicine permission numbers for Jinlong capsule, Huaier granule, Fufang Banmao capsule, Kanglixin capsule, Ganfule capsule, Huachansu capsule, Yadanzi oil soft capsule, Xihuang capsule, and Cidan capsule were Z10980041, Z20000109, Z52020238, Z20025075, Z20060389, Z20090944, Z20070062, Z61020121, and Z20063914, respectively. The TCM decoctions used in this study included the Fuzheng Jiedu Xiaoji Formula and some other TCM decoctions prescribed by certified Chinese medicine doctors. The ingredients, functional classification, usage, and dosage of TCMs are shown in Table S1.

### 2.4. Statistical Analyses

In this study, SPSS (version 26.0; IBM Corp, Armonk, New York, USA) was used for statistical analysis, and *P* < 0.05 indicated statistical significance. Categorical variables were expressed as frequency, and the chi-square test was used for comparison between the two groups. Continuous variables conforming to the normal distribution were represented by mean ± standard deviation, and the *t*-test was used for comparison between the two groups. Continuous variables conforming to the skewed distribution were represented by medians with interquartile range (IQR), and the Mann–Whitney *U* test was used for the comparison of variables between the two groups. Cox regression was used for univariate and multivariate analyses to identify independent factors affecting the survival of patients with PLC. Kaplan–Meier survival analysis was used to assess overall survival outcomes, and the log-rank test was used to compare differences in survival curves. A forest plot was generated using GraphPad Prism 8 (GraphPad Software, La Jolla, CA, USA) to compare the 3-year mortality hazard ratio (HR) between the TCM group and the control group.

## 3. Results

### 3.1. Factors Affecting the Survival of Patients with PLC

Before performing PSM, the impact of baseline indicators on the 3-year survival of patients with PLC was analyzed using Cox regression. After multivariate Cox regression analysis, TCM (adjusted hazard ratio (aHR) 0.359, 95% CI 0.292–0.441, *P* < 0.001), high A/G (aHR 0.617, 95% CI 0.446–0.855, *P*=0.004), high lymphocyte count (aHR 0.822, 95% CI 0.699–0.967, *P*=0.018), resection (aHR 0.189, 95% CI 0.103–0.346, *P* < 0.001), and minimal invasiveness (aHR 0.219, 95% CI 0.160–0.299, *P* < 0.001) were independent protective factors for the 3-year survival of PLC. By contrast, Child–Pugh class B (aHR 1.561, 95% CI 1.217–2.003, *P* < 0.001), high AFP (aHR 1.988, 95% CI 1.644–2.404, *P* < 0.001), portal vein tumor thrombus (aHR 2.152, 95% CI 1.620–2.859, *P* < 0.001), high neutrophil count (aHR 1.165, 95% CI 1.127–1.205, *P* < 0.001), high MELD score (aHR 1.049, 95% CI 1.024–1.074, *P* < 0.001), other causes (aHR 1.486, 95% CI 1.046–2.111, *P*=0.027), BCLC stage B (aHR 1.838, 95% CI 1.359–2.485, *P* < 0.001), BCLC stage C (aHR 1.624, 95% CI 1.104–2.390, *P*=0.014), and BCLC stage D (aHR 2.503, 95% CI 1.189–5.270, *P*=0.016) were independent risk factors (Table 1).

### 3.2. Baseline Characteristics of the Two Cohorts

Before PSM, 1,111 (59.8%) patients received TCM for ≥3 months during follow-up. Compared with the control group, the TCM group showed a higher rate of alcohol consumption, antiviral therapy, albumin, A/G, prothrombin activity, lymphocyte count, hemoglobin, Child–Pugh class A, BCLC stage A and B, resection, and minimally invasive. Moreover, the TCM group showed a lower rate of cirrhosis, decompensation, other causes, HBV-DNA ≥500 IU/ml, alanine aminotransferase, aspartate aminotransferase, total bilirubin, total bile acid, creatinine, INR, NLR, platelets, AFP ≥400 ng/ml, Child–Pugh class B and C, number of tumors ≥2, portal vein tumor thrombus, BCLC stage C and D, and palliative therapy than the control group. After PSM, there was a balanced performance between the two groups in terms of the major factors (Table 2).

### 3.3. Survival Analyses

**Figure 2:**
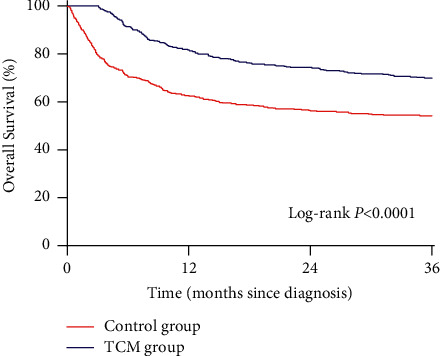
The 3-year overall survival rate of patients with primary liver cancer in TCM and control groups after propensity score matching.

**Figure 3:**
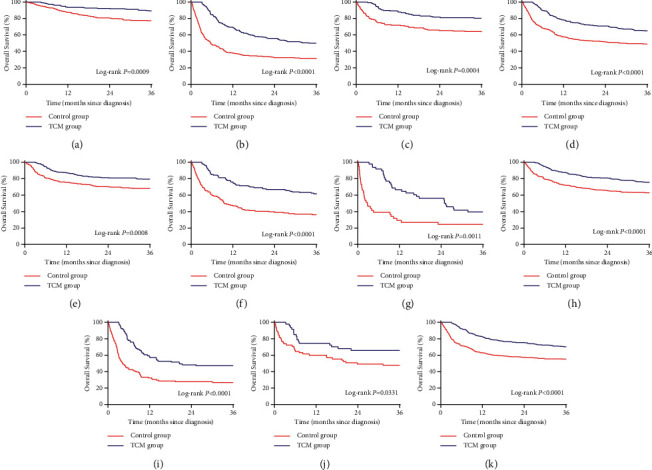
The 3-year overall survival rate of patients with primary liver cancer in different subgroups after propensity score matching. (a) BCLC staging A. (b) BCLC staging B–D. (c) Compensation period. (d) Decompensation period. (e) Child–Pugh class A. (f) Child–Pugh class B. (g) Child–Pugh class C. (h) AFP (ng/ml) ˂400. (i) AFP (ng/ml) ≥400. (j) Noncirrhosis. (k) Liver cirrhosis.

Before and after PSM, the 3-year overall survival rates were 33.3% and 54% in the control group and 79.7% and 69.7% in the TCM group, respectively. After PSM, the results showed that the overall survival rate of the TCM group during the 3-year follow-up was significantly higher than that of the control group (*P* < 0.0001; Figure 2). After PSM, the 3-year survival rates of patients with BCLC stage A and B–D were 88.7% and 50% in the TCM group and 77.2% and 31.1% in the control group, respectively; patients in the compensatory and decompensated phases were 80.3% and 64.6% in the TCM group and 63.5% and 49% in the control group, respectively; patients in Child–Pugh class A, B, and C were 79.3%, 60.9%, and 39.6% in the TCM group and 67.8%, 36.2%, and 24.4% in the control group, respectively; patients with AFP (ng/ml) <400 and ≥400 were 75.2% and 47.3% in the TCM group and 62.6% and 26.9% in the control group, respectively. Furthermore, patients with noncirrhosis and cirrhosis were 66% and 70.1% in the TCM group and 47.7% and 55% in the control group, respectively. Kaplan–Meier survival analysis showed that patients in the above subgroups had a higher 3-year overall survival rate in the TCM group than in the control group (*P* < 0.05; Figure 3).

### 3.4. Survival of Hepatitis B Virus-Related PLC

**Figure 4:**
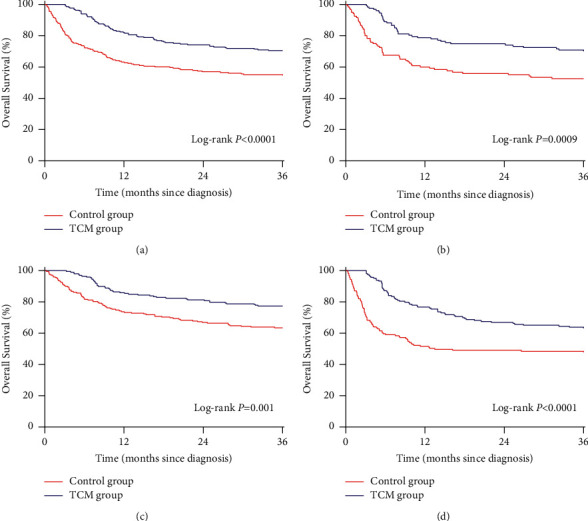
The effect of traditional Chinese medicine on the 3-year overall survival rate of hepatitis B virus-related primary liver cancer after propensity score matching. (a) HBeAg-negative. (b) HBeAg-positive. (c) Low-level HBV-DNA. (d) High-level HBV-DNA.

After PSM, the 3-year overall survival rates of patients with hepatitis B virus-related PLC in the HBeAg-negative and HBeAg-positive groups were 70.5% and 70.1%, respectively, in the TCM group and 54.7% and 52.5%, respectively, in the control group (Figures 4(a) and4(b)). The 3-year overall survival rates of patients with hepatitis B virus-related PLC in the low-level HBV-DNA (HBV-DNA <500 IU/ml) and high-level HBV-DNA groups (HBV-DNA ≥500 IU/ml) were 77.3% and 63.2%, respectively, in the TCM group and 63.4% and 47.8%, respectively, in the control group (Figures 4(c) and 4(d)). Whether HBeAg is negative or positive, and the patient's serum HBV-DNA is at a low or high level, the 3-year overall survival rate of patients with hepatitis B virus-related PLC in the TCM group was higher than that in the control group (*P* < 0.05).

### 3.5. Analysis of Mortality Risk

**Figure 5:**
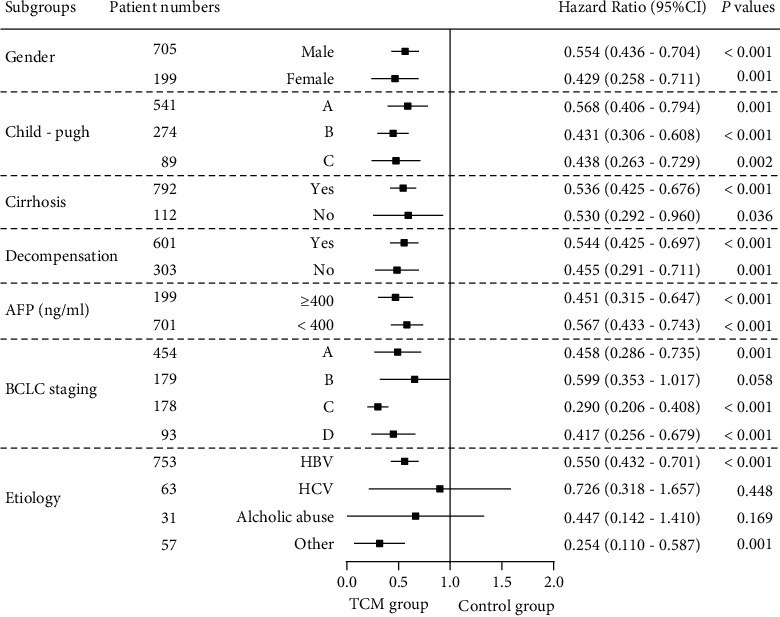
Subgroups analysis of the 3-year death risk of primary liver cancer in patients from traditional Chinese medicine and control cohorts.

According to the patient's sex, Child–Pugh class, liver stiffness, liver compensatory function, serum AFP level, BCLC stage, and etiology, patients with PLC were divided into multiple subgroups. The forest plot showed that after PSM, regardless of sex, Child–Pugh class, AFP levels, with or without cirrhosis, compensation or decompensation period, BCLC stage A, C, and D, hepatitis B virus or other causes, the TCM group exhibited a lower risk of death in 3 years than the control group (*P* < 0.05; Figure 5). There was no significant difference in the 3-year mortality risk of patients with BCLC stage B, hepatitis C virus, and alcoholic liver between the two groups. (*P* > 0.05; Figure 5).

## 4. Discussion

As an auxiliary and alternative therapy, TCM has a good effect on various cancers and few side effects and has gradually gained recognition. A meta-analysis of 1,941 patients with hepatocellular carcinoma showed that compared with Western medicine alone, adjuvant TCM treatment could increase the sensitivity of tumors to treatment, reduce adverse events, and improve the overall survival rate [14]. Liao et al. [15] showed that the risk of death in patients taking adjuvant TCM was significantly lower than that in patients who did not receive TCM. A prospective randomized controlled trial [16] indicated that TCM can improve the 5-year recurrence-free survival rate and overall survival rate of patients with small hepatocellular carcinoma compared with TACE. Previous studies have shown that TCM is beneficial for the prognosis of liver cancer, but few studies have analyzed the effect of TCM on the 3-year survival of large-scale PLC patients based on liver function, clinical liver cancer staging, and hepatitis B virus-related PLC. In this study, we found that for different Child–Pugh grades, early or advanced liver cancer, HBeAg-negative or -positive, and high or low serum HBV-DNA levels, patients with PLC treated with TCM had a better survival rate.

TCM compounds, Chinese medicine extracts, and single Chinese medicines have been widely used for the prevention and treatment of PLC [17,18]. Modern pharmacological studies have shown that the antitumor active ingredient in Jinlong capsule, the gecko sulfated polysaccharide-protein complex, at 100 and 200 *μ*g/ml, can significantly inhibit the proliferation of liver cancer SMMC-7721 cells [19]. Jinlong capsules may also improve the immune function of patients by increasing the percentage of CD3+, CD4+, natural killer (NK) cells, and the CD4+/CD8+ ratio in patients with hepatocellular carcinoma [20]. Li et al. [21] showed that Huaier polysaccharide (TP-1) is the main active component of Huaier granules, which inhibits the hypoxia-inducible factor (HIF)-1 alpha/vascular endothelial growth factor (VEGF) and RNA binding factor-1 (AUF-1)/astrocyte elevated gene-1 (AEG-1) signaling pathway and plays an antihepatic tumor and antimetastasis effect in liver cancer. Zhao et al. [22] showed that compared with TACE therapy alone, TACE combined with Fufang Banmao capsule could increase human CD3+ and CD4+ cells, and Fufang Banmao capsules could inhibit the proliferation of liver cancer cells by inhibiting the expression of the c-Myc gene and promoting the expression of the p53 gene. The eight Chinese medicines in the Kanglixin capsule have anticancer activity. Curcumin and emodin can inhibit the proliferation of tumor cells, promote their apoptosis, inhibit tumor angiogenesis, and improve the sensitivity of tumor cells to radiotherapy and chemotherapy [23]. Pan et al. [24] showed that Ganfule capsules can regulate phosphatidylinositol 3-kinase catalytic alpha (PIK3CA) and caspase-8 (CASP8) protein expression, thus indirectly affecting the PI3K-Akt/JAkt-STAT signal transduction pathway and inhibiting the proliferation and invasion of human liver cancer HepG2 cells. Huachansu capsules are widely used for the treatment of various tumors. With regard to antiliver cancer effects, the IgA production pathway of the intestinal immune network induced by APOBEC3F in tumor tissues is inhibited by bufalin, thereby preventing the proliferation of liver tumor cells [25, 26]. Bai et al. [27] showed that *Brucea javanica* oil blocks human liver cancer HepG2 and Huh7 cells in the G0/G1 phase of the cell cycle in vitro, thereby inhibiting the proliferation of liver cancer cells. Xihuang capsules can downregulate the expression level of VEGF and inhibit the level of matrix protease in liver tumor cells, thereby exerting an inhibitory effect on liver tumors [28]. Cidan capsules have a cytotoxic effect on liver cancer and inhibit the synthesis of cyclooxygenase-2 (COX-2) and VEGF in liver cancer cells, thereby reducing the recurrence and metastasis of liver cancer [29]. Research by our team [30] showed that the Fuzheng Jiedu Xiaoji formula can effectively inhibit the proliferation and migration of liver cancer cells by regulating the AKT/CyclinD1/p21/p27 pathway.

Our study has some limitations. First, although PSM was performed between the two groups to eliminate confounding factors, there were still differences in several baseline indicators between the two groups. However, we have matched the important factors. Second, in terms of treatment, because this article is a retrospective study, patients could decide by themselves whether to choose Chinese medicine treatment, hence some consequent deviations in our research. Finally, this study mainly discussed the therapeutic effects of TCM on the survival of patients with PLC. The effects of different types of Chinese patent medicines on the survival of patients with PLC were not compared.

## 5. Conclusion

The results of our study showed that TCM adjuvant therapy could increase the 3-year overall survival rate in patients with PLC. Adjuvant TCM may play a beneficial role in the clinical treatment of PLC. However, this conclusion requires further verification in randomized controlled trials that are prospective and large-scale.

## Figures and Tables

**Table 1:** Factors related to the 3-year survival of patients with primary liver cancer.

Variables	Univariate analysis			Multivariate analysis		
	HR	95% CI	*P* values	HR	95% CI	*P* values
TCM	0.173	0.148–0.203	<0.001	0.359	0.292–0.441	<0.001
A/G	0.129	0.103–0.162	<0.001	0.617	0.446–0.855	0.004
LC, 10^9^/L	0.555	0.486–0.634	<0.001	0.822	0.699–0.967	0.018
NC, 10^9^/L	1.243	1.218–1.269	<0.001	1.165	1.127–1.205	<0.001
AFP ≥400, ng/ml	3.303	2.842–3.839	<0.001	1.988	1.644–2.404	<0.001
PVTT	13.206	11.17–15.613	<0.001	2.152	1.620–2.859	<0.001
MELD score	1.144	1.131–1.156	<0.001	1.049	1.024–1.074	<0.001

*Child–Pugh class*						
A	Reference					
B	4.356	3.653–5.194	<0.001	1.561	1.217–2.003	<0.001
C	10.533	8.670–12.796	<0.001			

*BCLC stage*						
A	Reference					
B	2.403	1.843–3.134	<0.001	1.838	1.359–2.485	<0.001
C	16.80	13.551–20.828	<0.001	1.624	1.104–2.390	0.014
D	17.804	14.272–22.210	<0.001	2.503	1.189–5.270	0.016

*Type of treatment*						
Resection	0.036	0.023–0.057	<0.001	0.189	0.103–0.346	<0.001
Minimally invasive	0.059	0.049–0.070	<0.001	0.219	0.160–0.299	<0.001
Palliative	Reference					

*Etiology*						
HBV	0.716	0.595–0.861	<0.001			
HCV	0.973	0.730–1.297	0.852			
Alcoholic hepatitis	1.783	1.296–2.453	<0.001			
Other	1.624	1.215–2.170	0.001	1.486	1.046–2.111	0.027
ALT, U/L	1.002	1.001–1.002	<0.001			
TBIL, *µ*mol/L	1.005	1.005–1.006	<0.001			
Cr, *µ*mol/L	1.002	1.001–1.003	<0.001			
PTA, %	0.965	0.961–0.969	<0.001			
INR	1.345	1.258–1.438	<0.001			
NLR	1.065	1.057–1.072	<0.001			
RBC, 10^12^/L	0.602	0.551–0.657	<0.001			
Hb, g/L	0.984	0.981–0.986	<0.001			
PLT, 10^9^/L	1.002	1.001–1.003	<0.001			
Cirrhosis	1.854	1.454–2.365	<0.001			
Decompensation	3.534	2.918–4.282	<0.001			
Tumor number ≥2	3.520	2.998–4.133	<0.001			
Antiviral treatment	0.384	0.324–0.456	<0.001			
Male sex	1.152	0.959–1.383	0.129			
Age, years	1.012	1.005–1.020	0.001			
History of smoking	1.138	0.983–1.317	0.083			
History of alcohol use	1.176	1.016–1.361	0.030			
Family history of PLC	0.875	0.677–1.131	0.309			
Diabetes	1.256	1.064–1.483	0.007			
Hypertension	1.100	0.935–1.296	0.251			

HR, hazard ratio; TCM, traditional Chinese medicine; A/G, albumin/globulin; LC, lymphocyte count; NC, neutrophil count; AFP, alpha-fetoprotein; PVTT, portal vein tumor thrombus; MELD, model for end-stage liver disease; BCLC stage, barcelona clinic liver cancer stage; HBV, hepatitis B virus; HCV, hepatitis C virus; ALT, alanine aminotransferase; TBIL, total bilirubin; Cr, creatinine; PTA, prothrombin time activity; INR, international normalized ratio; NLR, neutrophil-lymphocyte ratio; RBC, red blood cell count; Hb, hemoglobin; PLT, platelets.

**Table 2:** Baseline characteristics of patients with primary liver cancer before and after propensity score matching.

Variables	Before propensity score matching				After propensity score matching		
	Control group (*n* = 748)	TCM group (*n* = 1111)		*P* values	Control group (*n* = 452)	TCM group (*n* = 452)	*P* values
Age, years	57 (50,63)	56 (50,62)		0.200	57 (50,64)	56 (50,62)	0.484
Gender (male/female)	582/166	874/237		0.659	346/106	359/93	0.297
Family history of PLC (yes/no/NA)	60/687/1	122/989/0		0.053	32/419/1	37/415/0	0.501
History of smoking (yes/no/NA)	333/413/2	498/608/5		0.809	193/257/2	207/243/2	0.643
History of alcohol use (yes/no/NA)	306/439/3	499/612/0		0.028	170/279/3	220/232/0	0.001
Diabetes (yes/no/NA)	165/581/2	250/860/1		0.633	101/350/1	95/356/1	0.889
Hypertension (yes/no/NA)	195/551/2	281/827/3		0.932	128/323/1	105/345/2	0.189
ALT, U/L	39.2 (25.4,65.5)	28.7 (19.9,45.3)		<0.001	33.2 (22.3,53.9)	28.5 (19.8,45.3)	0.003
AST, U/L	58.6 (32.7,127.3)	33.1 (24.2,51.1)		<0.001	37.9 (27.1,67.6)	37.9 (26.6,57.1)	0.093
TBIL, *µ*mol/L	27.3 (15.5,48.9)	16.7 (11.8,24.6)		<0.001	19.9 (12.5,32.0)	18.7 (12.8,28.8)	0.197
ALB, g/L	33.5 (28.8,38.9)	38.5 (33.4,42.2)		<0.001	36.4 (31.4,40.6)	36.8 (30.9,41.3)	0.632
A/G	1.0 (0.8,1.3)	1.3 (1.0,1.5)		<0.001	1.2 (0.9,1.5)	1.2 (0.9,1.4)	0.525
TBA, *µ*mol/L	22.4 (8.8,56.0)	14.5 (6.2,34.5)		<0.001	15.7 (7,42.6)	20.6 (7.7,48.3)	0.150
Cr, *µ*mol/L	67.6 (58,81.9)	67 (58.9,76)		0.028	67 (57.2,78)	66.9 (57.9,75)	0.218
PTA, %	70 (58.4,83)	82.8 (70,94)		<0.001	76.1 (64.5,89)	78 (65.4,91)	0.241
INR	1.2 (1.1,1.3)	1.1 (1.0,1.2)		<0.001	1.1 (1.0,1.3)	1.1 (1.0,1.3)	0.361
LC, 10^9^/L	1.0 (0.7,1.4)	1.2 (0.8,1.7)		<0.001	1.1 (0.7,1.6)	1.1 (0.7,1.6)	0.802
NLR	3.3 (1.9,5.7)	2.0 (1.4,2.9)		<0.001	2.4 (1.5,4.0)	2.2 (1.4,3.3)	0.013
Hb, g/L	124 (101.8,138.4)	134 (118.2,147)		<0.001	129.6 (109,142.2)	130.3 (112,144.3)	0.146
PLT, 10^9^/L	101.3 (66.4,152.2)	93.4 (61.3,146.1)		0.017	101.1 (62.3,149.5)	86 (59,139)	0.019
HBeAg (positive/negative/NA)	205/500/43	310/725/76		0.593	121/296/35	127/293/32	0.863
HBV-DNA, IU/ml (≥500/<500/NA)	327/389/32	384/691/36		<0.001	162/275/15	163/274/15	0.998

*Etiology*							
HBV	623/125	948/163		0.233	377/75	376/76	0.929
HCV	51/697	77/1034		0.925	33/419	30/422	0.695
Alcoholic hepatitis	26/722	42/1069		0.732	6/446	25/427	0.001
Other	48/700	44/1067		0.017	36/416	21/431	0.04
Antiviral therapy (yes/no/NA)	249/412/87	518/503/90		<0.001	199/205/48	188/221/43	0.552
Cirrhosis (yes/no)	678/70	899/212		<0.001	387/65	405/47	0.069
Decompensation (yes/no)	585/163	593/518		<0.001	296/156	305/147	0.526
Child-Pugh class (A/B/C)	278/285/185	816/235/60		<0.001	270/141/41	271/133/48	0.675
AFP, ng/ml (≥400/<400/NA)	257/481/10	184/925/2		<0.001	108/342/2	91/359/2	0.394
Tumor number (≥2/<2/NA)	430/289/29	387/689/35		<0.001	208/229/15	192/239/21	0.396
PVTT (yes/no/NA)	282/398/68	72/1010/29		<0.001	83/343/26	68/360/24	0.371
BCLC stage (A/B/C/D)	233/97/224/194	728/227/94/62		<0.001	224/91/94/43	230/88/84/50	0.749

*Type of treatment*							
Resection (yes/no)	32/716		162/949	<0.001	32/420	32/420	1.000
Minimally invasive (yes/no)	276/472		801/310	<0.001	276/176	284/168	0.584
Palliative (yes/no)	440/308		148/963	<0.001	144/308	136/316	0.565

ALT, alanine aminotransferase; AST, aspartate aminotransferase; TBIL, total bilirubin; ALB, albumin; A/G, albumin/globulin; TBA, total bile acid; Cr, creatinine; PTA, prothrombin activity; INR, international normalized ratio; LC, lymphocyte count; NLR, neutrophil-lymphocyte ratio; Hb, hemoglobin; PLT, platelets; HBV, hepatitis B virus; HCV, hepatitis C virus; AFP, alpha-fetoprotein; PVTT, portal vein tumor thrombus; BCLC stage, barcelona clinic liver cancer stage.

## Data Availability

The data used to support the findings of this study are available from the corresponding author upon request.

## Supplementary Materials

Supplementary Materials Table S1: description of the name, ingredients, functional classification, usage, and dosage of traditional Chinese medicine.
