# Supplementary material for: Effects of Traditional Chinese Medicine Adjuvant Therapy on the Survival of Patients with Primary Liver Cancer
Source: Evid Based Complement Alternat Med. 2022 Mar 17;2022:9810036. doi: 10.1155/2022/9810036 (PMC8947932; doi:10.1155/2022/9810036)
Supplement: Supplementary Materials — Table S1: description of the name, ingredients, functional classification, usage, and dosage of traditional Chinese medicine. [file 9810036.f1.docx]

Table S1: The name, ingredients, functional classification, usage, and dosage of TCM

| TCM name | Ingredients | Functional classiﬁcation | Usage and Dosage |
| --- | --- | --- | --- |
| Jinlong Capsule | Fresh *Bungarus multicinctus*, Fresh *Agkistrodon acutus* (*Guenther*), Fresh gecko | To make the blood stasis unobstructed and the nodules dissipated | 1g per time  Oral, 3 times a day |
| Huaier Granule | Fermentation products of *Trametes robiniophila Murr* | To enhance body resistance, to make the blood stasis unobstructed and the nodules dissipated | 20g per time  Oral, 3 times a day |
| Fufang banmao Capsule | Banmao (*Mylabris phalerata Pallas*), Renshen (*Panax ginseng*), Huangqi (*Radix astragali*), Ciwujia (*Acanthopanax senticosus*), Sanleng (stem of *Sparganium stoloni erum*, Buch. -Ham.), Banzhilian (*Scutellaria barbata*), Ezhu (*Curcuma phaeocaulis* Valeton), Shanzhuyu (*Fructus corni*), Nvzhenzi (*Fructus Ligustri Lucidi*), Fel Ursi powder, Gancao (*Glycyrrhiza uralensis* Fisch) | To break blood and eliminate blood stasis, to remove toxins and eliminate sores | 0.75g per time  Oral, 2 times a day |
| Kanglixin Capsule | Awei (Resin of *Ferula sinkiangensisK. M. Shen* or *Ferula fukanensis K.M.Shen*), Jiuxiangchong (*Aspongopus chinensis Dallas*), Dahuang (*Radix et Rhizoma rhei*), Jianghuang (*Rhizoma curcumae longae*), Hezi (*Fructus chebulae*), Muxiang (*Radix of* *aucklandia lappa Decne*), Dingxiang (*Flos caryophylli*), Dongchongxiacao (*Cordyceps sinensis*) | To strengthen the body resistance and eliminate the causing disease factors, to soften the hardness and dissipate nodules | 1-1.5g per time  Oral, 3 times a day |
| Ganfule Capsule | Dangshen (*Radix codonopsis*), Biejia (*Carapace* *of trionyx sinensis Wiegmann*, processed with Vinegar), Chonglou (*Rhizoma paridis*), Baizhu (*Rhizoma atractylodis macrocephalae*, fried), Huangqi (*Radix astragali*), Chenpi (*Pericarpium citri reticulatae*), Tubiechong (*Eupolyphaga sinensis*), Dahuang (*Radix et Rhizoma rhei*), Taoren (*Semen persicae*), Banzhilian (*Scutellaria barbata*), Baijiangcao (*Herba patrinia*), Fuling (*Poria cocos* (*Schw.*) *Wolf*), Yiyiren (*Coix lacryma*), Yujin (*Radix Curcumae*), Sumu ((*Lignum sappan*), Muli (*ostrea gigas thunberg*), Yinchen (*Artemisia capillaris Thunb*), Mutong (*Akebia Decne*), Xiangfu (*Cyperus rotundus L*), Chenxiang (*Aquilaria sinensis* (Lour.) Gilg), Chaihu (*Radix bupleuri*) | To invigorate the spleen and regulate qi, to remove blood stasis and soften the hardness, to clear away heat and detoxify | 3g per time  Oral, 3 times a day |
| Huachansu Capsule | Dry toad skin | Detoxification, swelling, and pain relief | 0.9g per time  Oral, 2 times a day |
| Yadanzi oil soft capsule | Brucea javanica oil, Soybean lecithin | To corrode wart, to soften and dissipate nodules, to clear away heat and detoxify | 2.12g per time  Oral, 2-3 times a day |
| Xihuang Capsule | Artificial bezoar, Artificial musk, Moyao (*Commiphora myrrha* (Nees) Engl.), Ruxiang (Resin of *boswellia carterii Birdw*.) | To detoxify and dissipate nodules, to reduce swelling and relieve pain | 1-2g per time  Oral, 2 times a day |
| Cidan Capsule | Ezhu (*Curcuma phaeocaulis* *Valeton*), Shancigu (*Cremastra　appendiculata*), Yadanzi (*Brucea javanica* (*Linn.*) *Merr*), Maqianzi powder (*Strychnos　nux-vomica* *L*, processed), Hive, Huangqi (*Radix astragali*), Danggui (*Angelica sinensis*), etc. | To remove blood stasis and detoxification, to reduce swelling and dispel lumps, to replenish qi and nourish blood | 1.35g per time  Oral, 4 times a day |
| Fuzheng jiedu xiaoji  Formula | Dangshen 15g (*Radix codonopsis*), Huangqi 15g (*Radix astragali*), Baizhu 15g (*Rhizoma atractylodis macrocephalae*), Fuling 15g (*Poria cocos* (*Schw.*) *Wolf*), Shashen 15g (*Adenophora stricta Miq*), Maidong 15g (*Ophiopogon japonicus*), Danggui 15g (*Angelica sinensis*), Shudi 15g (*Rehmanniae radix praeparata*), Ezhu 10g (*Curcuma phaeocaulis* Valeton), Qiye yizhihua 10g (*Paris polyphylla Smith*), Banxia 9g ((*Pinellia ternata* ( *Thunb*.) *Makino*) | To invigorate the spleen and nourish yin, to invigorate blood circulation to remove blood stasis, to soften the hardness and dissipate nodules, to clear away heat and detoxify | 1 dose per day  decoct in water and take orally 1 hour after breakfast and dinner |
